# Clinical audit on possible causes of hospital initiated clinic cancellations and recommendation to improve the service

**DOI:** 10.1192/bjo.2021.853

**Published:** 2021-06-18

**Authors:** Tanzida Haque, Mosab Mohammed Jodat Ibrahim, Bapu Ravindranath

**Affiliations:** 1 Mersey Care NHS Foundation Trust, Merseycare NHS Foundation Trust; 2 Mersey Care NHS Foundation Trust, Merseycare NHS Foundation Trust, HEE NW; 3 Mersey Care NHS Foundation Trust

## Abstract

**Aims:**

The aim of this audit is to explore the possible causes of clinic cancellation in an inner city CMHT and the recommendation to reduce the burden.

**Background:**

Cancellations of planned appointments have been a major and long-standing problem for healthcare organisations across the world. It represents a significant loss of revenue and waste of resources, have significant psychological, social and financial implications for patients and their families and represent a significant loss of training opportunities for trainees. Re-scheduling appointment is one of the major issues of inconvenience to the patients. It also increases workload for the patient appointment team.

**Method:**

Data have been collected retrospectively from patient appointment booking team regarding clinic cancellation with causes of cancellation recorded in the system (01/07/2019–30/09/2019). The investigators have investigated if the cancellation has been made when it was absolutely necessary to cancel the clinic (Unavailability of doctors due to leave/on calls) and if patients have been informed at least 8 weeks prior to the appointed clinic as per trust protocol.

**Result:**

Total number of 193 clinics were booked at the CMHT from July 2019 – September 2019. About 54% clinics were cancelled during the time period. The Clinic Cancellation rate was higher in September (68%) and was lowest in August (30.30%). As the month of July is the changeover period for trainees, the number of clinics booked during August was relatively less than normal. 72% clinics were cancelled by junior doctors and 28% clinics were cancelled by consultants at the CMHT. The major cause of clinic cancellation was unavailability of the junior doctors due to on call (31.58%) which was not communicated to the patient appointment booking team. Due to annual leave, 25% clinics were cancelled and 21% clinics were cancelled due to study leave. In both cases it is evident that, lack of communication between clinicians and patient appointment team are primarily responsible for hospital-initiated clinic cancellations. As per Patient Appointment booking team, around 50% cases, patients were informed 8 weeks in advance before cancelling the clinics.

**Conclusion:**

This is evident from this audit that the number of hospital-initiated clinic cancellations can be reduced by improving communication between Patient Appointment booking service, Medical staffing department and clinicians. The findings of the audit have been shared locally with CMHT managers, clinicians and with the patient appointment booking team.

